# The ability to learn new written words is modulated by language orthographic consistency

**DOI:** 10.1371/journal.pone.0228129

**Published:** 2020-02-13

**Authors:** Chiara Valeria Marinelli, Pierluigi Zoccolotti, Cristina Romani

**Affiliations:** 1 Department of History, Lab. of Applied Psychology and Intervention, Society and Human Studies, University of Salento, Lecce, Italy; 2 Department of Psychology, University of Rome La Sapienza, Rome, Italy; 3 ISTC Institute for Cognitive Sciences and Technologies, CNR, Rome, Italy; 4 Aston University, Birmingham, United Kingdom; Universiteit van Amsterdam, NETHERLANDS

## Abstract

**Introduction:**

It is well known that a difficulty in forming lexical representations is a strong predictor of reading and spelling difficulties even after controlling for the effects of other cognitive skills. Our study had two main interrelated aims. First, we wanted to examine whether the ability to learn new written words (lexical learning) varies as a function of the orthographic consistency of the language of the learner. Second, we wanted to evaluate the cognitive abilities involved in orthographic lexical learning and whether they differed as a function of language consistency.

**Method:**

163 Italian children and 128 English children performed a lexical learning task as well as tasks assessing several cognitive skills potentially related to the ability to establish orthographic representations.

**Results:**

We found that children learning an orthographic inconsistent orthography (English) were better able to learn novel *written* words presented in association with pictures than children learning a consistent orthography (Italian). This was true for both younger and older primary school children and also when children were matched for school grade. Lexical learning may be better in English children because the many irregularities of this language promote storing in memory whole-word representations and processing larger orthographic units. In Italian, instead, reading can be accomplished successfully on the basis of grapheme-phoneme conversion rules and on processing smaller orthographic units. This interpretation was supported by the pattern of cognitive skills associated with lexical learning skills in the two languages. Variations in lexical learning were explained by spatial visual memory and phonological awareness tasks in both languages, but phonological STM explained further variance in Italian, while a task tapping visuo-attentional capacity explained further variance in English.

**Conclusion:**

Learning a language with inconsistent orthography is associated with better lexical learning skills in children at different stages of primary school; the pattern of cognitive skills associated with lexical learning skills is also partially modulated by orthographic consistency.

## Introduction

“Lexical learning” is the ability to set up stable and accurate mental representations of words in the lexicon [1]. An associative learning process, *i.e*. the ability to create links between orthographic and phonological representations, plays a key role in learning to read, because this ability is fundamental in acquiring a sight vocabulary and in the development of orthographic representations [2]. Entries in the orthographic lexicon allow whole word recognition for fluent reading and correct productions in spelling. A difficulty in forming lexical representations would have negative consequences for accurate spelling [1, 3] and for fast and proficient reading, even in children of orthographically transparent languages [4, 5]. Some authors suggested that the impaired reading speed and poor spelling skills observed in dyslexic children in consistent orthographies may be primarily due to a dysfunction in storing word representations [6], with a consequent impoverished orthographic lexicon [7–10].

The ability of storing written representations has been the subject of extensive investigation through both implicit and explicit verbal learning tasks. In the former case, orthographic representations of pseudo-words are acquired incidentally while the child reads a text passage which contains them; in the latter case, the child is explicitly instructed to memorize pseudo-words presented in association to pictures. Lexical learning skills, the object of the present study, have often been investigated in dyslexic participants, while fewer studies have examined typically developing readers. Some studies have investigate lexical learning in typically developing children thought spoken learning task [see 11] and results highlight that this ability is a strong predictor of reading and spelling abilities, even after controlling for the effects of other cognitive skills [12]. A lexical learning deficit has been found in children with dyslexia [again in terms of spoken learning 13–14] and in adults with developmental dyslexia and dysgraphia with both spoken and written learning tasks [see 1, 15, 16, 17], despite normal performance on tasks requiring learning associations with non-orthographic stimuli (such as learning to associate one abstract shape with another or pictures with nonverbal sounds such as coughs, *e.g*., [14, 18, 19]). Romani and colleagues [1, 17] found that the ability to learn new written words in adults with dyslexia explained most of the variance in word reading and spelling over and above phonological skills. Deficits of spoken learning have also been reported in children with dyslexia learning consistent orthographies (for German-speaking children: [6, 20]; for Dutch-speaking children: [19]). In Italian, dyslexic children show a poor orthographic lexicon [7, 8, 9] and a slower rate of learning (as a reduction in reading RTs) following multiple presentations of novel words [21], but no study has yet explored lexical learning skills among typically developing children.

A poor lexicon may result from poor educational experiences. Clearly, limited print exposure may affect the extent and quality of lexical knowledge [22–23]. Measures of print exposure (based on recognition of book authors or titles) correlate with spelling and reading, and particularly with lexical knowledge assessed with irregular word reading [24; but see 25 for contradictory results]. Therefore, impoverished orthographic lexical representations may result from a high truancy rate, reduced attention at school, and low compliance in doing homework. However, several studies have also shown that dyslexic individuals are impaired even when learning opportunities are equated in the lab [e.g., 1, 3, 17, 21], ruling out that poor orthographic lexical representations are solely the result of lack of learning opportunities.

### 1 Lexical learning: Self-teaching hypothesis and role of orthographic consistency

Lexical learning plays a crucial role in inconsistent orthographies such as English, by allowing correct reading and spelling of irregular words [26]. However, lexical learning is important also in consistent orthographies for which grapheme-to-phoneme conversion is enough to ensure accurate reading and spelling of almost all words. In fact, access to stored lexical representation promotes a transition from alphabetic to orthographic strategies, leading to a fluent and fast reading.

Share [27] suggested that phonological decoding is a prerequisite to the establishment of orthographic representations. According to this hypothesis, working out the phonology of a new word allows the reader to understand the word and this, in turn, is the basis to establish a new orthographic lexical representation (see [28] for a review; see also [29–30] for recent attempts to implement the self-teaching hypothesis in computational terms). Relatively few (successful) exposures (four or even fewer) may be enough in young children to acquire word-specific information [10, 31–35], with progressively less dramatic changes on subsequent trials. According to this hypothesis, lexical learning should be easier in languages where lexical decoding is easier, that is in orthographically transparent languages.

Alternatively, however, ease of lexical learning may depend not or not only on ease of decoding, but more importantly on practice with orthographic processing. In inconsistent orthographies, the presence of irregular words may encourage children to rely on larger processing units which are more phonologically consistent and, ultimately, on stored representations [36–40]. Consistent results have been reported in studies examining reading errors [41–45] and the effects of psycholinguistic variables ([41, 46], see also [47, 48] for an English-Italian comparison on the same samples examined in the present study). In this case, you may predict the opposite: that lexical learning will be easier in orthographically opaque languages which practice processing of larger orthographic units. Results relevant to these different hypotheses have been mixed. Share [28] found that orthographic learning occurred earlier in children learning an inconsistent orthography (e.g., English) compared to children learning a consistent orthography (i.e., pointed Hebrew). On the other hand, Van Daal and Wass [49] compared orthographic learning in Danish and Swedish, two cousin languages which differ in grapheme-phoneme consistency. They found lexical learning skills were better in Swedish children learning the more consistent orthography. Furthermore, lexical learning skill were more strongly associated to reading and spelling in Swedish children, even after controlling for vocabulary and phonological working memory abilities.

### 2 Cognitive factors affecting lexical learning

Several different cognitive abilities may influence lexical learning and may contribute to generate possible cross-linguistic differences.

The quality of phonological representations may be important for both spoken and written lexical learning. This has been demonstrated by an association between tasks of lexical learning and tasks of phonological awareness [19, 50–51] and/or rapid automatized naming (RAN [6, 13]). A short-term record may also be necessary to build a corresponding long-term representation, as shown by associations between (spoken) lexical learning and phonological short-term memory (STM) in typically developing children and in adults ([12, 50]; for a review see [52]), as well as in dyslexic children [6] and adults ([1, 52]; see also [53] for a developmental phonological STM deficit associated with lexical learning difficulties). Finally, a proficient use of sublexical decoding may be important as suggested by the self-teaching hypothesis (see also [54]).

These findings, however, do not exclude that lexical learning (especially in a *written* form) may also tap independent skills related to the ability to establish a new representation in terms of new ordering of letters or sounds in a crowded lexical space. Several results suggest some independence between phonological and lexical learning abilities. Spoken lexical learning and phonological awareness predict *independent* reading variation in typically developing readers [6]. Moreover, lexical learning deficits are found in children and adults with dyslexia even in the absence of any impairment of phonological awareness or STM [for deficits of written learning see: 15, 55; for spoken learning see 20]. The case for independence is particularly strong in the case of written learning. Di Betta and Romani [1] found that phonological STM was strongly associated with *spoken* lexical learning, but not at all with *written* learning, at least in adults with dyslexia whose phonological skills were impaired. Moreover, Romani et al. [17] showed that written lexical learning and phonological abilities predicted different orthographic skills in adults with developmental dyslexia: lexical learning was a strong predictor of word reading and spelling, while phonological abilities (although impaired) predicted success only in pseudo-word processing (when prediction was within the dyslexic group). Altogether, these results indicate that phonological skills are necessary, but probably not sufficient for good lexical learning. Visual-attention is important for proficient reading and may be equally important for orthographic learning since the different letters making up the word need to be attended to. In fact, difficulties in processing sequences of visual units are common in dyslexic children and adults (*e.g*., [56–59]) and coexist with lexical learning impairments (see [60]). However, different types of visual skills may be important for orthographic learning.

Here, we will consider a) visuo-spatial memory; b) visuo-attentional capacity; and c) serial visual-attention.

***Visuo-spatial memory*** is the ability to remember visuo-spatial patterns. This capacity may be important to remember words as visual patters (e.g., see [3]).

***Visuo-attentional capacity*** refers to the ability to process several visual units at once, as a chunk of visual information. The metaphor of an attentional window has been used to describe this capacity. A good task to measure it is the full report task used Valois et al. (e.g., see [59]) where an unpronounceable string of letters is briefly presented on the screen and the participant must recall, in any order, as many letters as possible. This task/capacity is most used in reading familiar words where recognition may occur based on processing the word envelope and decoding a few letters. Development of this capacity would be responsible of an attenuation or disappearance of the length effect in word reading with increased reading proficiency. Consistent with a role in whole word reading, Valdois and colleagues found that a letter span task predicted variation in exception word reading in dyslexic children even after factoring out phonological skills (*e.g*., [61–64]), but the relationship with lexical learning has not yet been examined.

In contrast, ***visuo-serial attention***, is the capacity to allocate attention to the individual letters of a word occupying specific order position. It is required for binding together identity and positional information. The metaphor of **divided spot-lights** has been used to describe this capacity (e.g., see [58, 60]). The best way to measure it is through same-different tasks where the participant is asked to compare two strings of letters or symbols [60, 65, 66] or where he/she is asked to reconstruct the ordering of a set of letter/symbols after presentation of a string [67] This capacity is most used in reading unfamiliar words correct production crucially rely on identification of all letters and of letters in position. Crowding may be detrimental for this skill (e.g., see [68]). Impairments to this skill may also affects spelling accuracy which–in irregular orthographies—requires more than reading a correct memory for the letters in the word. Romani et al. [60] found correlations between array matching tasks and word reading and spelling tasks in a mixed group of adult participants with and without dyslexia (as well as within the dyslexic group), even after partialling out phonological skills. Correlations with lexical learning tasks, however, were limited to certain conditions (*e.g*., only when the array were made up strings of consonants and not with strings of non-alphanumeric symbols). In addition, Romani et al. [3] showed that, in adult participants with dyslexia, written lexical learning was impaired and related to a task tapping temporal ordering of visual symbols (measured by an order reconstruction task using series of sequentially presented Hindi or Japanese characters) and to visuo-spatial memory (measured with recognition of the same Hindi characters), but not to phonological skills.

Different computational models have stressed the contribution of either visuo-serial attention or visuo-attentional span to reading. The Multi-Trace Memory model by Ans et al. [69] stresses a visuo-attentional capacity which allows whole words to be processed at once (global processing mode). If not enough of this capacity is allowed words will be processed in a more analytic processing mode. This model, however, fails to recognize that an analytic processing mode requires its own resources. This has been recognized by the SERIOL model [70]which has stressed the need to focus attention on individual letters in a *serial* fashion in order to develop phoneme-grapheme conversion abilities in the initial phases of literacy acquisition and to read accurately novel stimuli at any point in time. If serial attention and attentional capacity are indeed different capacity linked to different mode of processing it is possible that they will be differently relied upon by Italian and English children in learning novel words because the type of orthography encourages more a global mode of processing in English and an analytic mode of processing in Italian.

In summary, although several studies have explored the association between phonological skills and spoken learning, fewer studies have explored associations with *written* lexical learning, and even fewer have explored the association with alternative cognitive skills such as different types of visual abilities. Moreover, we know of no study which has directly compared associations across orthographies with different degrees of consistency. If lexical learning is approached in different ways by English and Italian children, it is possible that it would be differently related to different skills. If English children rely more on a strategy which encodes larger chunks of visual information at once (the whole word in the limit), they may show a stronger association with tasks tapping visuo-attentional capacity. By contrast, if the Italian children rely more on sublexical processing, they may show a stronger association with phonological tasks and with tasks tapping visual serial attention which is important to partial out the orthographic strings into small subunits (letters in the limit) for phonological conversion.

### 3 The present study

The present research is part of a larger investigation aimed at examining cross-linguistic differences between the English and Italian orthographies in a developmental perspective. In previous reports, we have described differences in the acquisition of reading (see [47]) and writing (see [48]) in children between second and fifth grade. In this report, we focus on two main, interrelated questions.

First, we want to examine whether lexical learning skills vary as a function of the orthographic consistency of the language of the learner. Operationally, we asked Italian and English children to learn *written* pseudo-words in an explicit paired associate learning task, through corrective feedback. We administered the task in the written modality, since spelling requires learning more detailed representations [e.g., 71–75]. Moreover, several studies have shown that orthographic representations are better learned through spelling than reading [e.g., 75–77]. We examined two different developmental stages of literacy in order to evaluate whether or not early cross-linguistic differences diminish with the progress of reading and spelling acquisition (and increased reliance on lexical processing). It is possible, in fact, that cross-linguistic differences would be visible in younger children due to the better use of lexical strategies in English children and to the prevalent use of phonological recoding skills in the Italian children, but reduce or disappear in older children when even the Italian children start to rely on lexical processing [78–80].

Secondly, we examined the cognitive skills involved in orthographic learning and whether their contribution differ as a function of language transparency. We examined not only phonological abilities (such as phonological awareness, phonological STM and RAN), but also different visuo-attentional skills (visuo-spatial memory, visuo-attentional capacity, visuo-serial attention). Overall, a greater involvement of phonological skills was expected in the Italian sample, while a greater involvement of visuo-attentional skills was expected in the English sample, due to the greater need of lexical processing in this inconsistent orthography.

## Methods

### 1 Participants

Participants were 163 Italian children and 128 English children (although there were some missing data in the English sample for some cognitive tasks, for a maximum of 40 missing data in the whole English sample). Children were selected from local public primary schools (in England, in the Birmingham area and, in Italy, in the Rome and Naples areas) and were examined in in the course of January-May 2014. Children with neurosensory deficits or cognitive impairment (as assessed by Raven’s Coloured Progressive Matrices or CPM; [81]) were excluded from participation.

Ideally, we wanted to compare Italian and English children matched for age and educational level. However, this proved difficult since formal education starts earlier in England than in Italy; thus, children in the same grade are generally younger in England. Therefore, we run two types of comparisons: one in which we tried to match children as much as possible for age, allowing them to be in different grades, and one on a sub-sample in which we matched children for educational level (grade) as well for age. In the age-matched comparison, two developmental stages were considered: younger children were in third grade in England and in second grade in Italy; older children were in fifth grade in England and fourth grade in Italy. The grade-matched comparison was limited to 5^th^ grade children. A new, smaller group of 5^th^ grade Italian children (N = 19) was selected form a sample of 29 Italian 5^th^ grade children, in order to compare to a subgroup of 19 5^th^ grade English children (selected from older English cohort) matched for age and Raven performance. No grade level match comparison could be carried out for the younger group due to funding limitations.

The demographic characteristics of our samples are shown in Table 1.

**Table 1:** Demographic characteristics of the groups of participants involved in the study.

**Age-matched comparison**						
	**Younger cohort**			**Older cohort**		
	**English**	**Italian**	Diff E-I	English*	**Italian**	Diff E-I
Grade	3rd	2nd		5^th^	4th	
N	58	58		70	86	
Gender	30M/28F	29M/29F	.85	32M/38F	40M/46F	.92
Age	7.9	7.3	< .001	10	9.6	< .001
Raven: raw accuracy (and z score according to normative data)	29.1 (z = .73)	22.8 (z = .43)	< .001 .06	31.4 (z = -.28)	29.3 (z = -.01)	< .001 .12
**Grade matched comparison**						
	**English**	**Italian**	Diff E-I			
Grade	5^th^	5^th^				
N	19	19				
Gender	10M/9F	9M/10F	.75			
Age	10.3	10.4	.07			
Raven: raw accuracy (and z score according to normative data)	31.4 (z = -.30)	31.0 (z = -.03)	.15 .37			

Despite our best efforts, the children in the age-matched comparison still differed in age, with the English children being older by 5–6 months. In England, in order to be admitted at school, children need to have reached the required age before the beginning of the school year (by August-September), whereas, in Italy, they can be younger as they only need to reach the required age by the end of the school year (end of April). This difference is responsible for the difference in age. Consistent with being slightly older, the English children had higher raw scores on the Raven CPM test, but differences disappeared when standardized scores according to normative data were used.

Parents were informed about the research activities and authorized their child’s participation by signing the appropriate informed consent. The study was reviewed and approved by the Ethics Committee of the IRCCS Fondazione Santa Lucia—Rome (Prot. CE-PROG.480) before the study began, as well it was approved by the local University committees and by the school authorities. The research was conducted according to the principles of the Helsinki Declaration. Participants were tested individually in a silent room of the school.

### 2 Lexical learning task

**Fig 1:**
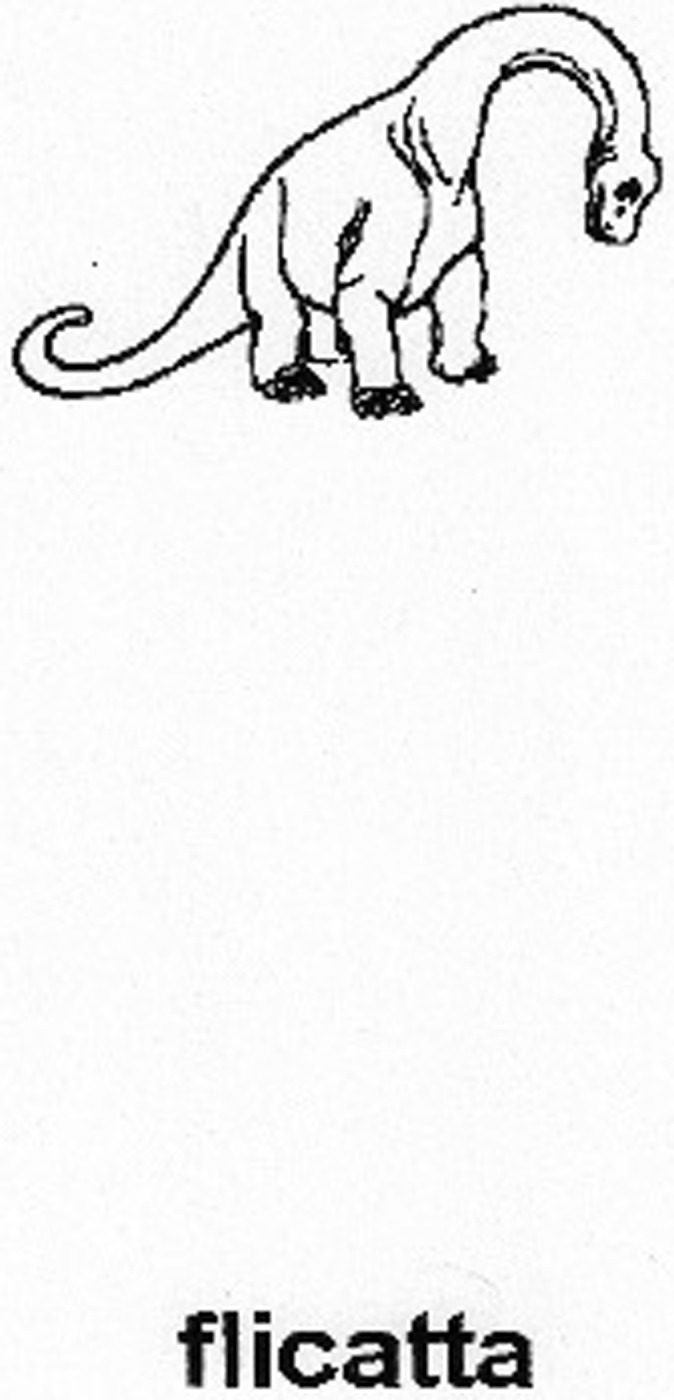
Example of card used in the lexical learning task.

Children were asked to learn new orthographic lexical representations by learning written pseudo-words in association with pictures (see Fig 1).

#### Materials

The pictures were black and white drawings from the International Picture Naming project [82–83]. The pictures in the two languages were the same.

The pseudo-words were constructed to have no clear resemblance to any word in either language. They were derived from real words by changing phonemes, but maintaining the original syllabic structure. Mean length was 6.85 letters. Pseudo-words were phono-tactically legal with relatively common grapheme-phoneme mappings; thus, they could be memorized by relying on both a phonological and/or an orthographic record. Note that, given the nature of the English orthography, even though English pseudo-words had unambiguous reading, they could still be spelled in different phonologically plausible ways if the participant used a less common rendition of the grapheme-phoneme mappings. Phonologically plausible errors were not possible in Italian given the highly regular nature of the orthography.

The pseudo-words associated to the pictures were matched in the two languages for: 1) number of phonemes, 2) number of syllables, 3) CV structure; 4) bigram frequency (English: MCWord database, [84]; Italian: Colfis database, [85]; in both cases reported to 1 million occurrences), 5) N-count (*i.e*., the number of words that can be obtained by changing one letter in the word and preserving the positions of the other letters; respectively from MCWord and Colfis database), and 6) frequency of the original words (according to the Colfis and Celex databases, in both cases reported to 1 million occurrences; all F < 1). Care was taken to avoid any semantic/phonological similarity between the pseudo-words and the names of the associated pictures. Stimuli are reported in S1 Appendix. Each pseudo-word was written below the picture in Times 24 italic font (see Fig 1).

The task was administered in one of two versions depending on the age of the participant: the younger children (7–8 years old) were given a shorter version with 6 picture-pseudo-word pairs, while the older children (at least 9–10 years old) were given a version with 9 picture-pseudo-word pairs. In the grade-level matched comparison the same version (i.e., the longer one) was used in both languages.

#### Procedure

On a first pass (introductory trial), the cards were presented to the children one by one with both the picture and the associated pseudo-word in sight. Participants were asked to look at the card carefully for 5 seconds; then the pseudo-word was covered up and they were asked to write it down. When they had finished, feedback was provided by removing the mask of the pseudo-word. Children were generally correct, with a near ceiling performance in both languages, but, if they made a mistake, they tried again. Note that children were not required to read pseudo-words aloud, because we wanted to encourage orthographic encoding and minimize the role of phonological memory; silent reading allows for the same amount of orthographic learning as reading aloud (e.g. [86–87]).

After the introductory trial, there were five learning trials (with cards presented in the same order in all trials) where the pictures were presented without the associated pseudo-word (due to the application of a mask on the pseudo-word) and the children were asked to remember and spell the pseudo-words without prompting. Feedback was provided by removing the mask of the pseudo-word to allow checking. In the case of a mismatch, the child had to copy the pseudo-word down. If the child recalled all the items correctly before the last learning trial, the task was discontinued and performance on the remaining trials was assumed to be correct. Thus, children were given five learning trials with the exception of cases where the task was stopped earlier because the child correctly spelled all the pseudo-words of a leaning trial.

#### Scoring

One point was scored for each pseudo-word spelled correctly. The score for each child was the percentage of pseudo-words spelled correctly across the five learning trials.

Additionally, we analysed the types of errors made. Errors were classified into: i) omissions: no response or attempt to spell the target; ii) related errors: a pseudo-word sharing at least 50% of letters with the target; iii) unrelated errors: a pseudo-word sharing less than 50% of letters with the target; iv) association errors: different pseudo-word from the learning set; v) association error with a misspelling: a recognizable (but incorrectly spelled) different pseudo-word from the learning set; vi) lexicalisations: a real word error; vii) related fragments: a response (consisting of 3 letters or less than 3 letters) sharing the letters with the target; viii) unrelated fragments: a partial response (= < 3 letters) not sharing letters with the target.

### 3 Cognitive tasks

Closely matched versions of tasks were used with the Italian and English children. For each task, the same or comparable stimuli (*i.e*., balanced for several critical psycholinguistic variables) were used in the two languages. All tasks were preceded by a short practice. Computerized tasks were administered with the e-prime 2 software [88]. When RTs were measured, we excluded: 1. RTs corresponding to errors, 2. self-corrections and wavers; 3 invalid responses due to technical problems; and 4. outliers (i.e., RTs below 250 ms or exceeding the individual mean ± 3 standard deviations). Accuracy was measured in percentage of errors (with lower scores for better performance as in the case of RTs). Due to the large number of tasks, each child was examined in four or five testing sessions, each session lasting about one hour. The following tasks were used.

#### Decoding skill

As part of a larger investigation, children were given a task involving reading single pseudo-words [48]. This allowed us to assess an association between decoding skills and Lexical learning. The task included 40 pseudo-words of increasing length (4, 5, 6 and 7–9 letters; N = 10 for each length) created from high frequency words by changing one to three letters. The sets were matched across languages for articulation point of the first phoneme and ortho-syllabic difficulty, such as the presence of double consonants, consonantal clusters and need for contextual rules ([89]see S2 Appendix). Stimuli were presented randomized on a portable computer. Each trial began with a fixation point that remained on the screen for 500 ms. Subsequently, a word appeared in the same position and remained on the screen until the child responded. The child was seated ca. 60 cm from the computer screen and was requested to read the stimulus as quickly and accurately as possible. The onset of the vocal response (vocal RTs) were recorded using a voice key (S-R Box). Errors were scored online but also tape-recorded for later checking. The mean of percentage of errors and RTs were computed for each child, as well as an index of Pseudo-word reading fluency, calculated as the number of pseudo-words read correctly over the total reading time.

#### Non-verbal reasoning

This was assessed using the Raven Coloured Progressive Matrices (CPM) with standard instructions [90]. The percentage of errors was computed for each child. Moreover, individual z scores were computed according to normative data in order to individuate cases with pathological performance (that were excluded from the sample, as reported in Participants section).

#### Phonological awareness

This was measured using a *Spoonerisms* and a *Phoneme deletion task*. Typically, tasks of phonological awareness are performed very well by readers of consistent orthographies (e.g., [91]) and this may limit their predictive power. For this reason, we used two tests (Phoneme deletion and Spoonerisms) less susceptible to ceiling effects which have been demonstrated to be sensitive to individual differences in children and adults [42, 92–95], as well as in young children [96].

In the *Spoonerisms task*, the child had to listen to 24 pairs of words and to exchange the first sounds of the two words in order to report the resulting stimuli (for example, “pala-sera” generates “sala-pera”). Twelve pairs resulted in real words and twelve did not. Scoring was based on the number of errors with one point awarded for each incorrect pair, and ½ point if only one of the words of the pair was correct. Self-corrections were counted as errors. The total score for each child was reported to the total number of pairs, in order to compute the percentage of errors.

In the *Phoneme-deletion task*, the child was given a spoken by-syllabic word and one of its phonemes. He was asked to repeat the word without the target phoneme. For example; “repeat ‘table’ without /t/”. The test had forty trials. After phoneme deletion, twenty stimuli resulted in real words and twenty in pseudo-words. For both sets, there were five items for each position in the word to delete (from 1st to 4th position). Stimuli were presented in a quasi-random order. The child was encouraged to think in terms of sounds, not letters. One point was scored for each incorrect trial. Self-corrections were counted as errors. The percentage of errors was computed for each child.

#### RAN

This was assessed with the colours and letters matrices developed by De Luca, Di Filippo, Judica, Spinelli and Zoccolotti [97]. Children were asked to name aloud the colours or letters as fast and as accurately as possible, row by row, from left to right. Performance was scored in terms of total time (number of seconds) or each condition.

#### Phonological STM

This was measured by repetition of series of pseudo-words and by a digit span test.

In the *Pseudo-word repetition task*, 10 lists of three bi-syllabic (five phonemes) pseudo-words were read aloud at a pace of about one every two seconds. The child was asked to repeat each list as accurately as possible. Each incorrect pseudo-word was awarded a point out and the percentage of errors was computed over a maximum score of 30.

In the *Digit span task*, the child was asked to repeat in the same order of presentation a list of digits read aloud by the experimenter at a pace of one every two seconds. The task started with lists of four digits and progressed with longer lists. Ten lists were presented for each length. The task ended when the child could not recall more than two lists of a given length. If the child made any mistake with the four-digit lists, three-digit lists were presented as well. Otherwise, scoring assumes that performance with lists of two and three digits was all correct. For scoring, each correctly recalled sequence was assigned 0.1 point. Thus, each length (which has ten lists) contributes one point to the span if all lists are recalled correctly.

#### Visuo-attentional capacity

This was assessed with a *Letter span task* (similar to that used by Bosse et al.’s task [62]). A fixation point was displayed for 1000 ms, followed by an array of five consonant letters (e.g., R H S D M) displayed simultaneously for a brief time (200 ms) at the centre of a computer screen. Each letter was used 10 times and appeared twice in each of the five positions. Letters were presented in uppercase (font Geneva 24) in black on a white background. No consonant was repeated within a given sequence. To minimise lateral masking, the inter-letter distance was 1 cm. Keeping the child at a distance of 60 cm, the array subtended an angle of approximately 3.8°. The child was asked to recall as many consonants as possible in any order. The task included twenty trials. The test administrator recorded the response of the child by typing the letters. RTs were not recorded. One point was scored for each letter recalled correctly, independently of the order of report. For each child, the total score was the sum of scores over the 20 trials. Also in this case, the children percentage of errors was computed (N of wrong letters/100 letters).

#### Visuo-serial attention

This was assessed with an *Array-matching task* (similar to that used by Romani et al. [60]) and an *Order-reconstruction task* (similar to that used by Romani et al. [3, 55]). Both of these tasks have a serial, ordering component and are less amenable to phonological decoding than the letter span task. In the *Array-matching task*, the child had to decide whether two sequences of five non-alphanumeric characters were the same or different. There were two conditions. In the Identity condition, the identity of a character was changed in the “different” trials; in the Order condition, the position of two characters was exchanged. The sequences used a pool of 24 characters taken from 12 different exotic fonts unknown to the participants. The aim was to have characters which were distinct from our alphabetic fonts and not easily nameable, but similar in terms of visual complexity. The identity condition consisted of 60 experimental trials (30 where the two sequences were the same and 30 where the two sequences were different). Each of the five positions was changed six times. The order condition consisted of 48 experimental trials: 24 same and 24 different. Only adjacent characters were swapped. There were six trials for each of the four possible order swaps. At the beginning of each trial, a central fixation point appeared for 1000 ms followed by a blank screen for 500 ms. Subsequently, two sequences were presented one next to the other. They remained on the screen until the participant made a choice. The child had to press the “m” key (labelled ‘same’) to indicate “same” and the “v” key (labelled ‘different’) to indicate “different”, using the two index fingers. Feedback was given after each trial. RTs (in ms) and errors (1 point for each incorrect response) were recorded.

The *Hindi order-reconstruction task* requires reconstructing the order of sequences of Hindi characters. The stimuli are presented either simultaneously or sequentially. Thus, the sequential version of the task particularly taps the ability to reconstruct temporal order, while the simultaneous version particularly taps serial attention (the ability to attend to stimuli as a function of their position). Our previous research has shown that the sequential condition of this task is more related to lexical learning abilities than the simultaneous condition, at least in dyslexic participants (see [55, 67]), because the sequential condition is better able to tap serial attentional skills needed for lexical learning. Therefore, here we include also the simultaneous condition as a control condition, to replicate previous results. Each trial started with a central fixation point presented for 1000 ms followed by a blank screen for 500 ms. Then, a sequence of four Hindi characters was displayed. In the *simultaneous* condition, they appeared together in a line. In the *sequential* condition, they appeared one after the other, at the centre of the screen, so that each character was replaced by the following one. In the simultaneous condition, the sequence remained on the screen for 3200 ms. In the sequential condition, each character remained on the screen for 800 ms. Immediately after presentation of the sequence, the child had to recreate the order of the characters using a set of tiles corresponding to the characters which were just presented. Ten sequences of four characters were constructed from two sets of 40 pseudo-Hindi characters (sets A and B). The same sets were used for the sequential and simultaneous presentations. Blocks of 5 sequences alternated between sequential and simultaneous presentations. For example: 1. Sequential A, 2. Simultaneous A, 3. Simultaneous A, 4. Sequential A; break; 1. Simultaneous B, 2. Sequential B, 3. Sequential B, 4. Simultaneous B. The order was counterbalanced across participants. The percentage of errors in each condition was computed.

#### Visual memory

This task was assessed using the materials of the Hindi order-reconstruction task (*Hindi Recognition task*). After a sequential and a simultaneous block were performed with a given set of characters, a recognition task was carried out in which the child was asked to recognize previously viewed characters from novel ones. The characters were presented on the computer screen one at the time. Each block involved 20 characters: 20 old and 20 new. The child had to press a key labelled ‘yes’ if he recognized the character, or a key labelled ‘no’ otherwise. The same procedure was repeated with the second set of characters. Order of presentation of sets of characters was counter-balanced across participants. Percentage of errors were computed for each child.

## Results

### 1 Comparing lexical learning in English and Italian children

**Fig 2:**
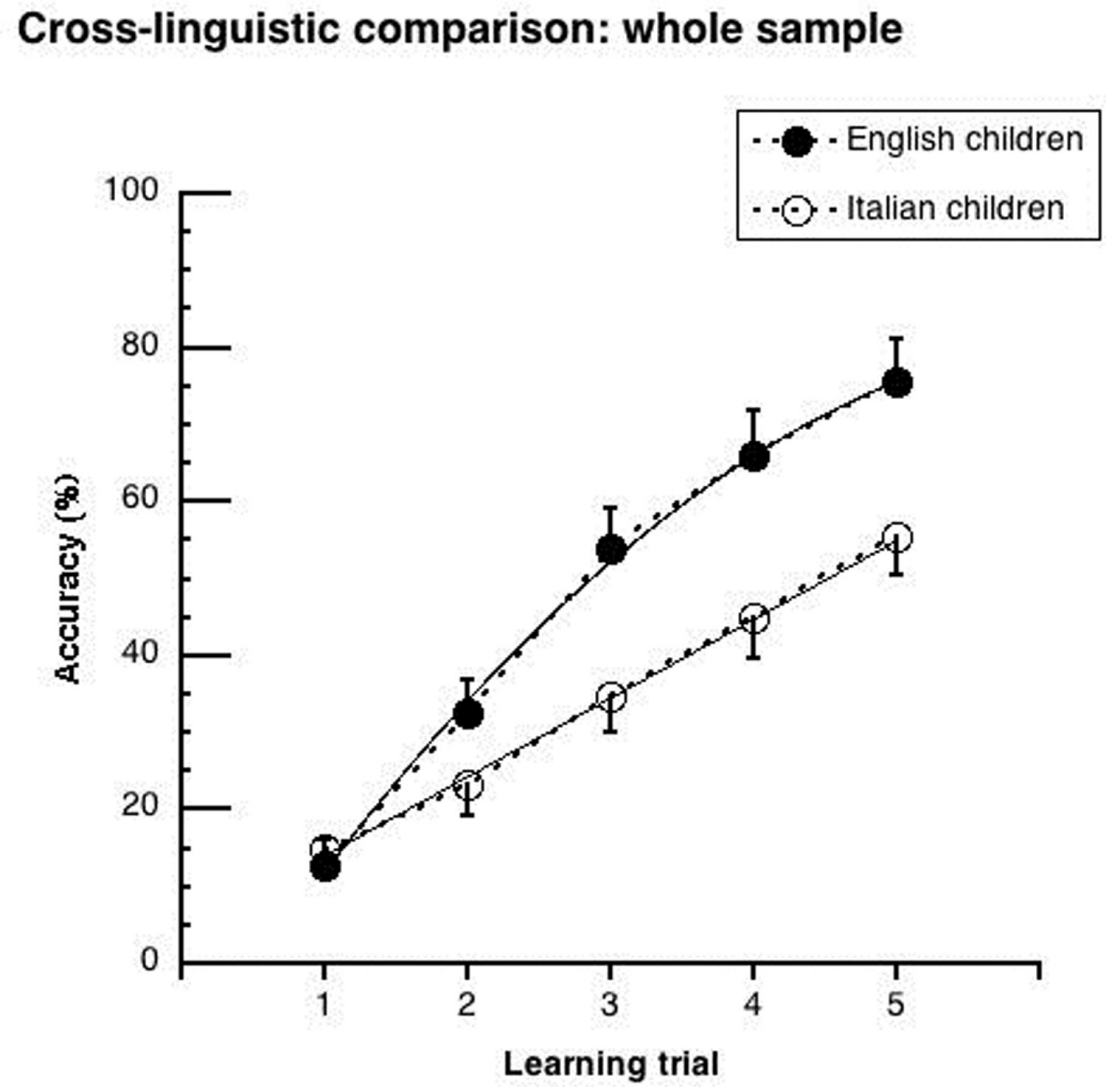
Mean performance (and .05 confidence intervals) on the lexical learning task as a function of trial sequence. Data (averaged between younger and older children) are presented separately for Italian and English participants.

**Fig 3:**
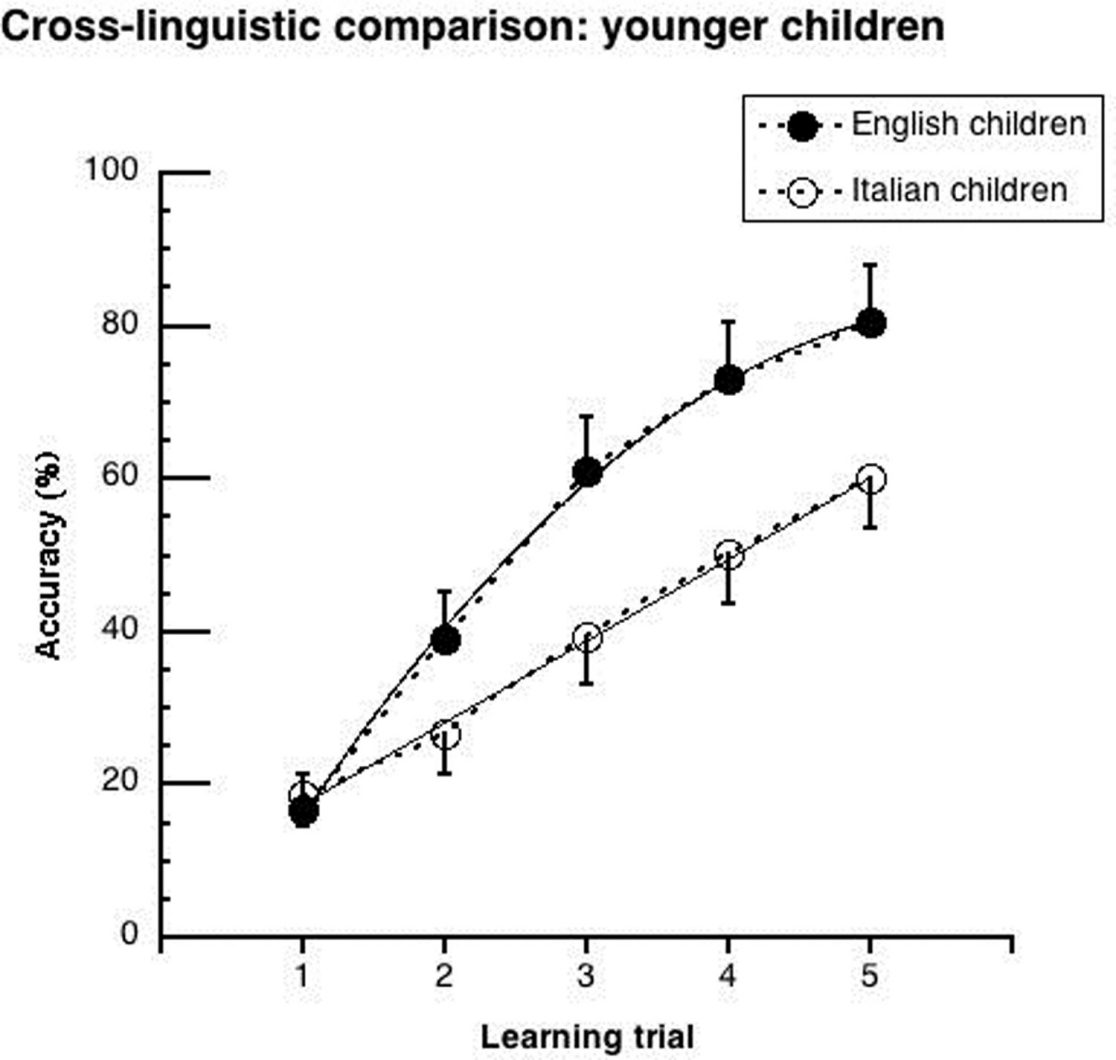
Mean performance (and .05 confidence intervals) of “younger” children on the lexical learning task as a function of trial sequence. Data are separately presented for Italian and English participants. Interpolation is based on significant trends. For the Italian children, a linear regression well explains the data (y = 0.75 + 9.88x; r^2^ = .99). For the English children, performance is well explained by the combination of a linear and a quadratic trend (y = -14.59 + 24.02x -1.39x^2^; r^2^ = .99).

**Fig 4:**
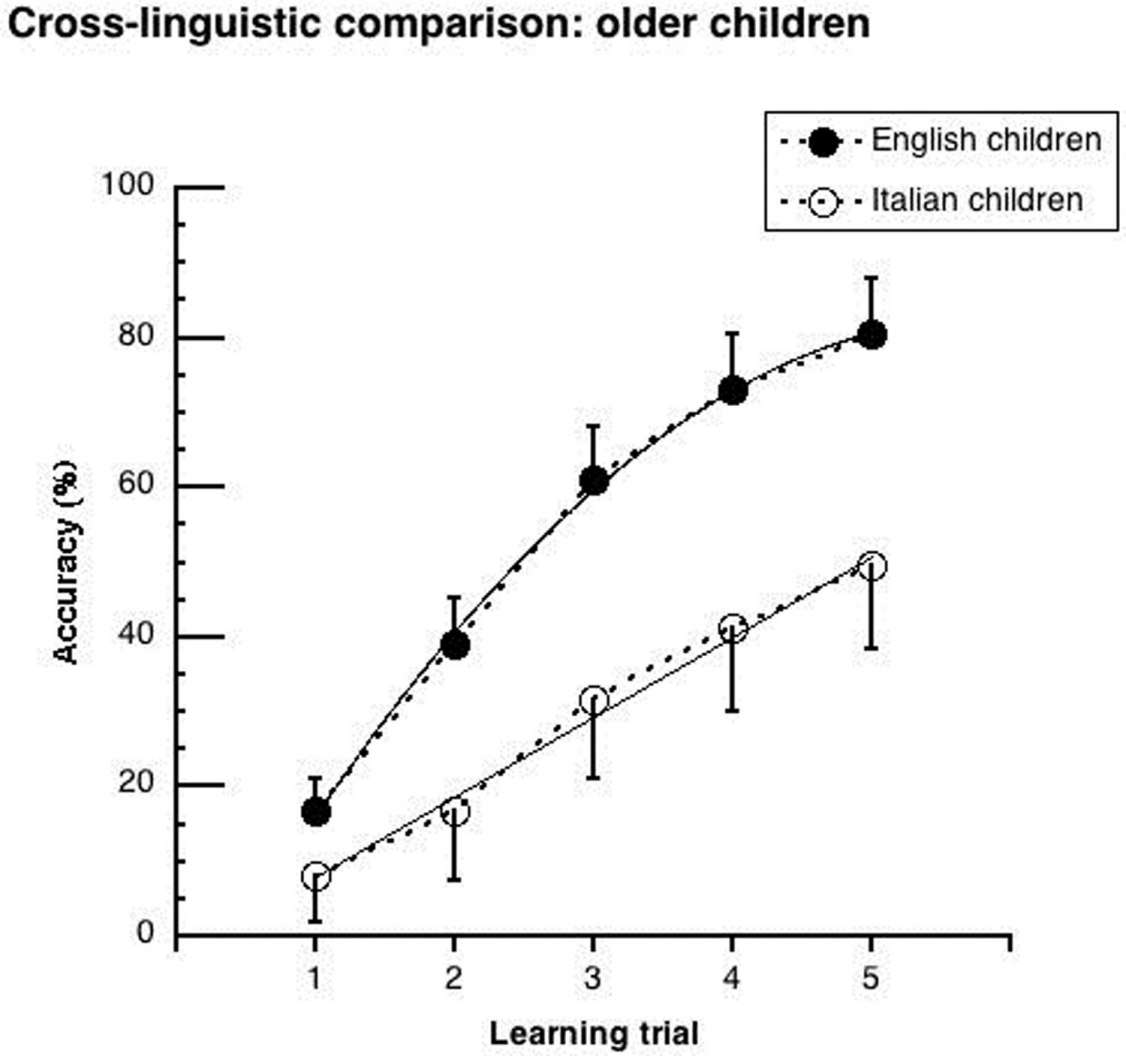
Mean performance (and .05 confidence intervals) of “older” children on the lexical learning task as a function of trial sequence. Data are separately presented for Italian and English participants. Interpolation is based on significant trends. For the Italian children, a linear regression well explains the data (y = 6.95 + 10.67x; r^2^ = .99). For English children, performance is well explained by the combination of a linear and a quadratic trend (y = -14.15 + 33.07x -2.82x^2^; r^2^ = .99).

**Fig 5:**
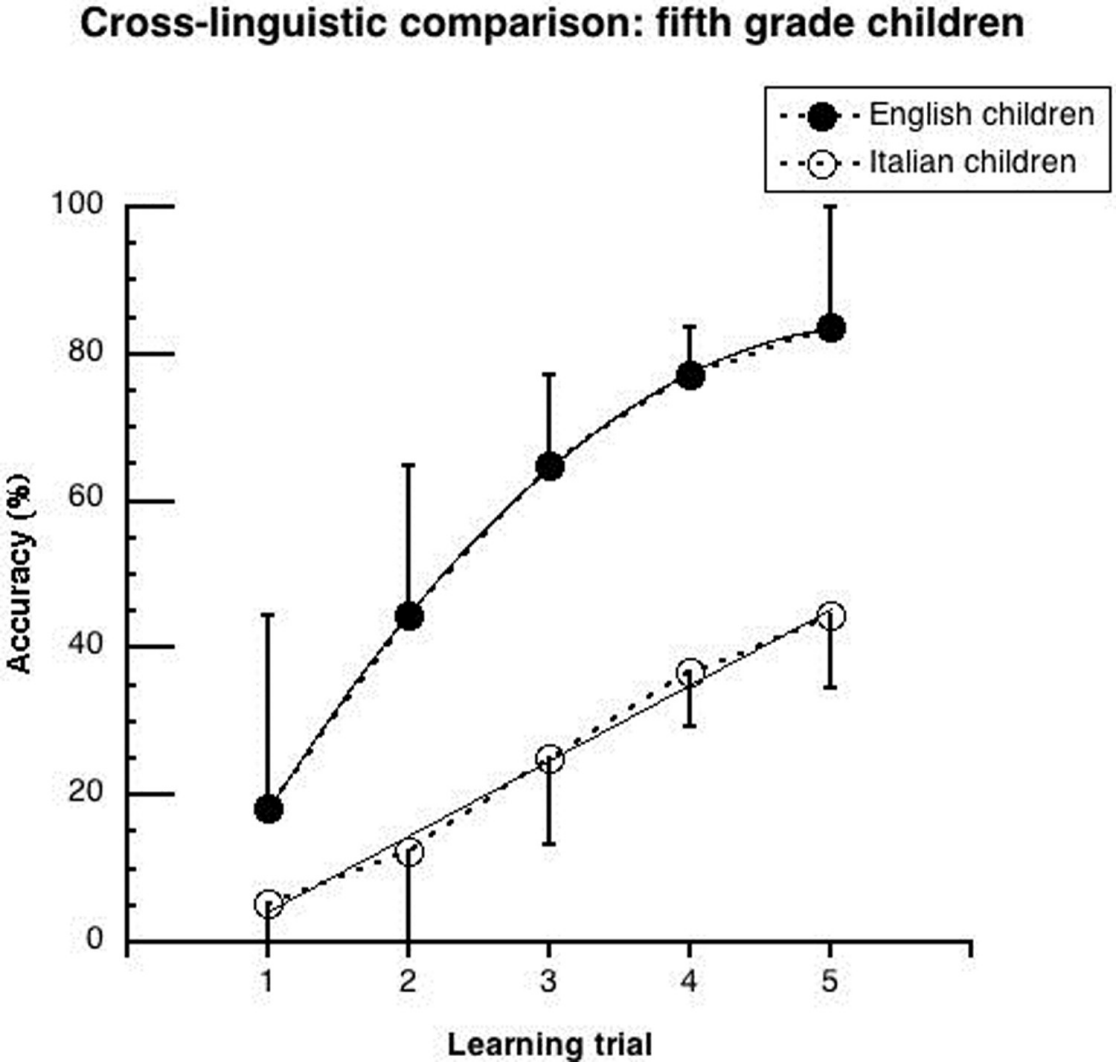
Mean performance (and .05 confidence intervals) of 5th grade Italian and English children on the lexical learning task as a function of trial sequence. Interpolation is based on significant trends. For the Italian children, a linear regression well explains the data (y = 10.29x - 6.08; r^2^ = .99). For the English children, performance is well explained by the combination of a linear and a quadratic trend (y = -3.43x^2^ + 36.93x - 15.44; r^2^ = .99).

Figs 2–5 show the mean performance in the lexical learning task for English and Italian children (Fig 2: results for groups, averaging between younger and older children; Figs 3 and 4: results for younger and older children, respectively; Fig 5: results for groups matched for schooling). In all cases, performance is initially similar for the two language groups but improves faster in the English cohorts.

For some children, testing was discontinued before the last trial (at the 4^th^ trial) because performance reached ceiling. Their proportion was 6.9%, 10.5% and 6.9% in the 2nd, 4th and 5th grade Italian samples, respectively. It was 25.9% and 40% in the 3rd and 5th grade English samples, respectively.

#### Age-matched comparison

We analysed differences between languages in lexical learning skills through ANOVA, with mean accuracy as the dependent variable, *language* (Italian/English) and *age cohort* (younger/older) as between-subjects factor, and *learning trial* (one to five learning trials) as a repeated measure. Due to the differences in age and Raven CPM performance between the two languages, analyses were replicated controlling for the effect of age and performance on the Raven CPM test (using scores standardized by age) with an ANCOVA. Significant interactions were explored by means of planned comparisons. In the grade-matched comparison, children were matched for school level (5^th^ grade). Thus, here, only the *language* and *learning trial* were considered.

The ANOVA showed significant main effects of *language* (F _(1,258)_ = 20.43, p < .0001; η^2^_p_ = .07), *age cohort* (F _(1,258)_ = 11.91, p < .001; η^2^_p_ = .04) and *learning trial* (F _(4,1032)_ = 479.80, p < .0001; η^2^_p_ = .65). Performance was better for the English than the Italian children (48.1% vs. 34.7%), for the older than the younger cohorts (46.5% vs. 36.3%), and for the later than the earlier trials (65.5% vs. 13.8%). The *language* by *learning trial* interaction was significant (F _(4,1032)_ = 27.42, p < .0001; η^2^_p_ = .10). As it can be seen in Fig 2, Italian and English children started with a similar level of accuracy at the first trial (about 15%), but the English children improved more over trials: they outperformed the Italian children by 9%, 19%, 21% and 20% at the 2nd, 3rd, 4th and 5th learning trial, respectively (at least p < .01). While the Italian children improved their performances by about 10% at each learning opportunity, the English children improved twice as much at the 2nd and 3rd learning trials (Δ = 20% and 21% respectively; at least p < .0001). They improved similarly to the Italian children at the 4th and 5th trials (Δ = 12% and 9%, respectively). None of interactions with *age cohort* were significant (*age cohort* by *learning trial*: F _(4, 1032)_ = 0.91, p = .46; *age cohort* by *languag*e: F _(4, 1032)_ = 0.32, p = .57; *age cohort* by *learning trial* by *language*: F _(4, 1032)_ = 0.52, p = .72), indicating similar cross-linguistic differences for the younger and older cohorts.

When *age* (in years) and *Raven* performance were entered in the analysis as covariates, the effects of the covariates were not significant (F about 1). The main effects of *language* (F_(1,235)_ = 4.26, p < .05; η^2^_p_ = .02) and the *language by learning trial* interaction (F_(4,940)_ = 8.16, p < .0001; η^2^_p_ = .03) were still significant.

#### Grade-matched comparison

Also in the grade-matched comparison, cross-linguistic differences in lexical learning skills were analysed through ANOVA, with mean accuracy as the dependent variable, *language* (Italian/English) as between-subjects factor, and *trial* (one to five learning trials) as a repeated measure. Significant interactions were explored by means of planned comparisons.

The ANOVA showed significant main effects of *language* (F_(1,36)_ = 23.16, p < .0001; η^2^_p_ = .07) and *learning trial* (F_(4,144)_ = 82.37, p < .0001; η^2^_p_ = .66). The English children performed better than the Italian children (57.7% vs. 24.8% respectively). Accuracy grew with increasing learning trials (from 11.7% to 64.0%). The *language* by *learning trial* interaction was significant (F_(4,144)_ = 6.15, p < .0001; η^2^_p_ = .09). As shown in Fig 5, at the first learning trial the English children were 12.9% more accurate than the Italian children (p < .05). This advantage increased to 32.2% at the 2nd learning trial (p < .0001) and to about 40% at the 3rd, 4th and 5th trials (at least p < .0001). An increase in accuracy as a function of learning trials was present for both the Italian (at least p < .01) and the English (at least p < .0001) children. However, the Italian children showed an accuracy increase of about 10% at each new learning trial while the English children showed a greater increase of 26.3% at the 2nd learning trials (p < .001), and similar increases to the Italian children at the 3^rd^, 4th and 5th learning trials (Δ = 20.5%, 12.3% and 6.4%, respectively).

#### Trend analyses

In the case of significant *learning trial* by *language* interactions, trend analyses were carried out to describe the learning curves of the different cohorts in each language (see Figs 3–5). These analyses assess both differences in the rate of acquisition and the possible presence of a plateau in the learning curve (i.e., through the presence of a significant quadratic trend).

Table 2 shows results separately for the younger and older cohorts as well as for the matched 5^th^ graders (for coefficient values please refer to the legends of Figs 3–5). In the Italian children, only a linear trend was significant. In the English children, there was both a linear and a quadratic trend in all comparisons.

**Table 2:** Trend analyses on data of the lexical learning task. Younger cohort: Italian 2^nd^ grader and English 3^rd^ graders. Older cohort Italian 4^th^ graders and English 5^th^ graders.

	Linear		Quadratic	
**Italian**	F	p	F	p
Younger cohort	117.1	< .0001	0.4	.55
Older cohort	202.4	< .0001	0.2	.67
5th graders	43.0	< .0001	0.0	1.00
**English**				
Younger cohort	268.6	< .0001	7.7	< .01
Older cohort	268.6	< .0001	38.6	< .0001
5th graders	108.8	< .0001	20.4	< .0001

#### Error analysis

We examined the type of errors in the Lexical learning task as a function of language, through the independent sample Kruskal-Wallis test.

Table 3 reports the percentage of each error type for both orthographies. In both languages, almost all incorrect responses were omissions or incorrect attempts to produce the target pseudo-word. The great majority of incorrect attempts was related to the target, sharing at least 50% of letters. Association errors (i.e., where children spelled or tried to spell a pseudo-word associated with another picture) were generally few, as were lexicalisations and fragments. The only significant cross-linguistic difference was in the number of related errors, i.e., in the ineffective attempt to retrieve the target pseudo-word, which was higher in the Italian than in English children (X^2^ = 13.04, p < .0001). Note that related errors for the English children included also phonologically plausible errors. These errors were not possible with the Italian stimuli. There are only very few possibilities for ambiguous spellings in the Italian orthography and these were not present among our stimuli.

**Table 3:** Type of errors at the lexical learning task.

Error type	Italian children		English children	
	Mean %	SD	Mean %	SD
Omissions	29.8	19.49	32.8	22.19
Related errors	13.9	7.67	9.6	10.08
Unrelated errors	3.1	5.37	1.9	5.81
Association errors (correct spelling)	2.0	3.37	1.9	4.33
Association errors (with misspelling)	5.4	7.17	2.6	4.99
Lexicalisations	1.1	2.27	0.5	1.19
Related fragments	0.2	0.61	1.4	5.16
Other fragments	0.3	0.98	0.6	3.08

### 2 Brief summary of results and comments

Both age- and school level-comparisons revealed that English children found it easier than Italian children to acquire new words in association with pictures. In the age-comparison, groups started with a similar level of performance, but the English children improved more across later learning trials. This pattern did not interact with age; *i.e*., it was evident in both the younger and older cohorts. Results were confirmed when age and Raven performance were entered as covariates in the analyses. When children were matched for years of schooling, the English sample performed better from the first trial and showed a larger increase in accuracy at each successive learning trial. In both age- and schooling-comparisons, trend analyses indicated a steeper linear regression for the English than the Italian children.

Some children were able to spell correctly all pseudo-words before the last learning trial; proportions were higher among the English children, again showing that they found the task easier. Error analyses showed a similar profile in the two groups, except for a higher percentage of related errors sharing more than the 50% of letters with the target in the Italian sample. Error patterns indicated that most of difficulties stemmed from poor recall of the exact pseudo-word rather than from difficulties with associations.

### 3 Cognitive factors affecting lexical learning

#### Correlations

In order to examine the cognitive skills involved in lexical learning, Pearson correlations between performance in the Lexical learning task and other cognitive tasks were carried out for each language. These analyses used participants’ z scores, computed on the basis of means and standard deviations of the respective age and language reference group (see Table 4 for raw means and SDs). This allowed collapsing data for the different age groups within each language. For cognitive skills for which more than one test was used, we computed an index averaging z scores on the relevant tests. A phonological awareness index was computed by averaging performance on the Spoonerisms and Phoneme deletion tasks; A RAN index by averaging naming times in the colour and letter RAN tasks; the Phonological STM index by averaging Pseudo-word Repetition and Digit span tasks; a Serial visual attention/order encoding index by averaging performance on all the conditions of the Array-matching task (in terms of RTs and errors) and of the sequential condition of the order-reconstruction task.

**Table 4:** Means and standard deviations (SD) for the lexical learning task, as well as the raw scores for all other cognitive tasks. For all measures in the table (except Digit span) higher scores correspond to worse performance (i.e., higher percentage of errors or longer RTs).

	AGE COMPARISON				AGE COMPARISON			
	younger cohorts				older cohorts			
	English		Italian		English		Italian	
	(3rd grade)		(2nd grade)		(5^th^ grade)		(4th grade)	
	mean	sd	mean	sd	mean	sd	mean	sd
Lexical learning task: % accuracy	**42.14**	*24.41*	**30.4**	*24.2*	**54.05**	*24.14*	**38.97**	*22.24*
Pseudoword reading fluency (N correct/RTs)	**.02**	.*01*	**.02**	.*01*	**.04**	.*01*	**.03**	.*01*
Pseudowords reading: RTs (ms)	**1740**	*1209*	**2560**	*1615*	**1267**	*866*	**1405**	*666*
Pseudowords reading: % errors	**28.95**	*21.67*	**26.65**	*18.00*	**14.83**	*14.13*	**10.64**	*8.16*
Raven: % errors	**19.5**	*11.39*	**37.26**	*15.17*	**12.58**	*9.18*	**18.57**	*11.57*
Ran Letters: sec	**89.42**	*25.42*	**111.66**	*25.08*	**66**	*14.30*	**88.53**	*20.15*
Ran Colours: sec	**113.84**	*41.02*	**100.64**	*18.01*	**83.83**	*20.61*	**80.84**	*14.69*
Spoonerism: % errors	**35.52**	*27.25*	**53.07**	*27.53*	**17.1**	*23.03*	**27.14**	*22.25*
Phoneme deletion: % errors	**26.57**	*23.33*	**26.69**	*17.02*	**16.12**	*23.17*	**12.09**	*10.25*
Repetition of pseudowords series: % errors	**45.59**	*21.28*	**59.17**	*20.16*	**27.42**	*15.79*	**35.19**	*20.64*
Digit Span	**4.75**	*0.54*	**3.99**	*0.60*	**5.62**	*0.78*	**5.09**	*0.59*
Visuo-attentional span: % errors	**32.96**	*13.99*	**28.48**	*11.50*	**21**	*13.87*	**28**	*17.82*
Array-matching—Identity: % errors	**11.14**	*10.83*	**5.72**	*6.41*	**5.8**	*5.97*	**3.82**	*5.03*
Array-matching—Identity: RTs (ms)	**5501**	*2482*	**6551**	*1373*	**4294**	*790*	**4285**	*1747*
Array-matching—Order: % errors	**10.51**	*11.06*	**4.96**	*5.88*	**5.34**	*6.61*	**5.54**	*9.63*
Array-matching—Order: RTs (ms)	**5409**	*2449*	**5697**	*1311*	**4049**	*693*	**4388**	*1412*
Order reconstruction—Hindi simultaneous: % errors	**47.03**	*13.08*	**57.37**	*14.02*	**29.2**	*18.16*	**44.01**	*15.05*
Order reconstruction—Hindi sequential: % errors	**48.84**	*13.72*	**60.73**	*12.03*	**33.72**	*17.46*	**42.67**	*12.55*
Hindi recognition: % errors	**32.13**	*12.19*	**30.6**	*12.24*	**23.53**	*10.29*	**26.93**	*11.63*

Table 5 shows correlations between the performance on the Lexical learning task and all the other tasks. The correlation coefficients of the English and Italian samples were compared using the Two Correlation coefficients test [98].

**Table 5:** Pearson correlations between the Lexical learning task and other cognitive tasks.

	Italian	English	Difference	
Task		Z score	p	
**Pseudoword reading fluency (correct/RTs)**	-.30 **	-.47 **	1.47	.14
RTs	.31 **	.32**	-.13	.90
Errors	.30 **	.37 **	-.51	.61
**Raven** (Errors)	**.10**	**.10**	.07	.95
**Phonological awareness index**	.43 **	.46 **	-.39	.70
Spoonerism (Errors)	.39 **	.43 **	-.45	.66
Phoneme deletion (Errors)	.38 **	.44 **	-.55	.58
**RAN Index**	.24 **	.34 **	-.84	.40
RAN letter (sec)	.16 **	.19 *	-.20	.84
RAN colour (sec)	.24 **	.36 **	-.97	.33
**Phonological STM index**	.39 **	**.10**	2.38	p < .05
Repetition of pseudo-words series (Errors)	.38 **	**.12**	2.15	p < .05
Digit span	-.32 **	**-.12**	1.57	.12
**Visuo-attentional span**	**.00**	.46 **	-4.14	p < .0001
**Visuo-serial attention index**	**.04**	**-.09**	1.01	.31
Array-matching order (Errors)	**.06**	.20 *	-1.04	.30
Array-matching order (RTs)	.17 *	**-.08**	1.90	.06
Array-matching identity (Errors)	**.15**	**.12**	.25	.80
Array-matching identity (RTs)	.19 *	**-.04**	1.77	.08
Order reconstruction Hindi Sequential (Errors)	.21 **	**.10**	.88	.38
**Order reconstruction Hindi Simultaneous** (Errors) (Errors)	**.08**	**.01**	.52	.60
**Visual memory**				
Hindi recognition (Errors)	.20 **	**.16**	.25	.80

* p < .05;

** p < .01. Differences between the two languages are assessed with the two-correlation coefficients test.

Pseudo-word reading correlated with lexical learning significantly in both languages and to a similar extent. Phonological tasks also correlated significantly with lexical learning. However, while phonological awareness and RAN were similarly associated with lexical learning in the two languages consistent with previous studies [99], correlations with STM tasks were stronger in Italian. Visual tasks also showed different associations in the two languages. Visuo-attentional capacity was correlated with lexical learning in English but not in Italian.

The pattern of correlation among the tasks tapping visuo-serial attention was more variable, but the visuo-serial attention index showed a stronger association with lexical learning in Italian than in English. Replicating previous results, the simultaneous condition of the order-reconstruction task showed smaller, non-significant correlations with lexical learning compared to the sequential condition. In fact, as predicted the order-reconstruction task showed a correlation with lexical learning only whether presentation of the stimuli was sequential condition highlighted the importance of serial attention and order encoding for lexical learning. Accuracy with the Hindi recognition task showed a similar association with the lexical learning in the two languages, also if the correlation reached the significance only in Italian sample.

#### Multiple regression analysis

We carried out a regression analysis (with the enter method) with the scores in the Lexical learning task as the dependent variable and the scores in the other cognitive tasks as predictors. Language and interactions between language and cognitive measures were also entered together as predictors to assess whether associations differed in the two languages. The following cognitive predictors were entered in the regression analysis (alone and in interactions with language): 1. Pseudo-word reading fluency; 2. Phonological awareness index; 3. RAN index; 4. Phonological STM index; 5. Visuo-attentional capacity; 6. Visuo-serial attention index; 8. Errors in the Hindi recognition task (as a measure of visual memory). The Raven scores and the simultaneous condition of the order reconstruction task were not added in the model, due to the absence of correlation between these tests and lexical learning in both languages. Inter-correlations between these predictors are reported in the S3 Appendix.

The results of the regression analysis are shown in Table 6. Lexical learning was predicted by phonological awareness (F_(1,244)_ = 7.07, p < .01) and visual memory (F_(1, 244)_ = 4.28, p < .05), with higher ability being associated with better lexical learning. *Language* was not significant *per se* (F_(1, 244)_ = 2.60, p = .11), but interacted with phonological STM (F_(1, 244)_ = 5.27, p < .05) and visuo-attentional capacity (F_(1, 244)_ = 5.22, p < .05): Phonological STM (Coeff. = 0.34; t = 2.30, p < .05) was more strongly associated to lexical learning in Italian than in English; the opposite was true for the Visuo-attentional capacity (Coeff. = -0.34; t = -2.28, p < .05).

**Table 6:** Results of regression analysis with the lexical learning scores as dependent variable, and language and the scores in the other cognitive tasks as predictors. Note that, in the case of Language and interactions with Language, the coefficient refers to Italian (taken as reference language). I. = Index.

	F_(1,244)_	P	B coefficient	T	P
MODEL (r^2^ = .29)	6.42	< .0001			
Intercept			-.05	-.56	.58
Language	2.60	.11	.18	1.61	.11
Pseudo-word reading fluency	1.84	.18	-.12	-.89	.37
Phonological Awareness I.	7.07	< .01	.23	1.58	.12
Ran I.	1.46	.23	.13	.91	.36
Phonological STM I.	.19	.66	-.14	-1.25	.21
Visuo-attentional span	.45	.50	.22	1.72	.09
Serial Attention/Order Encoding I.	.01	.91	-.13	-.78	.44
Visual Memory	4.28	< .05	.19	1.97	.05
Language x Pseudo-word reading fluency	.03	.85	.03	.19	.85
Language x Phonological Awareness I.	.01	.93	.02	.09	.93
Language x Ran I.	.02	.88	-.03	-.16	.88
Language x Phonological STM I.	5.27	< .05	.34	2.30	< .05
Language x Visuo-attentional span	5.22	< .05	-.34	-2.28	< .05
Language x Serial Attention/Order Encoding I.	1.90	.17	.28	1.38	.17
Language x Visual Memory	1.20	.28	-.13	-1.09	.28

### 4 Brief summary of results and comments

Pseudo-word reading fluency was related to lexical learning skills to a similar extent in both languages. Furthermore, phonological awareness and RAN tasks were robustly correlated with our (written) lexical learning task in both languages. This is expected since all these tasks measure the quality of phonological and orthographic representations which is crucial to learn new orthographic representations (see for example [100–102]).

The regression analysis showed that the Phonological Awareness index predicted the largest share of variance in lexical learning in both languages. The RAN index did not explain any further variance after the Phonological Awareness index, consistent with these tasks sharing a high proportion of variance. The Hindi recognition task tapping visual memory contributed significantly to explain variance in the lexical learning task without any interaction with language. These results indicate that, in both orthographies, the ability of store lexical representations is related to visual memory as well as to phonological awareness.

Importantly, there were also differences between the two languages. Phonological STM was associated with lexical learning in Italian, but not in English. It significantly correlated with it and, in the regression analysis, made a further contribution to explain variance. Visual skills also showed different patterns of association. In Italian, lexical learning tended to correlate more with tasks tapping visuo-serial attention, while in English it correlated more with the visuo-attentional capacity. Taken together these differences suggest that Italian and English children approach the task of learning new orthographic representations differently. This will be examined more thoroughly in the Discussion.

## Discussion

Our study reports two key findings: 1. English children are more efficient at lexical learning than Italian children; 2. The two languages show both similarities and differences in the cognitive abilities associated with lexical learning skills. We will review and discuss these findings in turn.

### 1 Better lexical learning acquisition in English than Italian children

English children showed a faster pace in learning new written words than Italian children, across younger and older cohorts. English and Italian groups started with a similar level of performance, but the English children improved faster. It is important to keep in mind that English children start literacy acquisition in school a year before the Italian children. Thus, their better lexical learning skills may be a consequence of more prolonged schooling. However, the same results held when children were matched for school level. The English children still performed better, in this case from the first learning trial. Moreover, while all children steadily improved with each further learning opportunity (yielding a linear function), the English children showed initially a steeper increase and then a plateau (marked by a quadratic trend). This pattern indicates that the Italian children required more learning trials to reach a stable performance.

Overall, these findings are in keeping with the idea that orthographic learning varies with the orthographic depth of the language and it is more effective in more inconsistent orthographies [28]. Several reasons might be responsible for the lower performance in the Italian children. The most likely possibility is the presence of many irregularities in the English orthography which encourages processing larger orthographic units and promotes storing in memory whole-word representations [36–38, 103].

The present investigation was part of a larger study in which we examined cross-linguistic differences in reading [47] and spelling [48] between the same group of English and Italian children. In spelling, the English children showed lower accuracy and larger frequency and regularity effects [48]. In reading, they showed stronger frequency and lexicality effect, but a reduced length effect and faster reading times. These results are consistent with the use of larger processing units in the English children which may increase reading speed, but at the cost of reduced accuracy in both reading and spelling. The lexical strategy of the English children was more error-prone (at least in these initial acquisition phases) presumably because of the greater difficulty posed by the irregular English orthography. In Italian, instead, the highly consistent orthography allowed good performance without memorization of orthographic patterns. Therefore, the delay of the Italian children in the use of a lexical reading strategy—demonstrated by the analyses of the psycholinguistic variables—go hand in hand with our experimental results of a delay in the acquisition of orthographic lexical representations in the laboratory.

We expected an earlier acquisition of lexical processing in the English than in the Italian children because the inconsistency of the English orthography encourages processing of larger orthographic units (due to their higher consistency respect to smaller units [39–40]). We expected differences to diminish or disappear in older children when lexical processing becomes more prevalent also among the Italian children, as demonstrated by better spelling of irregular words [78] and by a better ability to recognize phonologically plausible errors [79] in 4^th^ grade. Instead, the English children showed better lexical learning in both younger and older cohorts. It is possible, however, in fact likely, that any difference would have reduced or disappeared if still older children were tested.

Our results diverge from those of van Daal and Wass [49] who reported better lexical learning in Swedish children learning a more consistent orthography than in Danish children learning a more irregular orthography. We do not have a good explanation for this discrepancy. This difference could be mediated by the fact that the Swedish children were better spellers and readers than the Danish children [49]. However, in our study, the Italian children were also better at decoding than the English children, but, in spite of this, showed worse learning. It is possible that the Swedish children were better in the initial leaning trials, but did not improve as fast as the Danish children (differences in pace of learning were crucial in our study). As van Daal and Wass [49] did not separately examine performance across learning trials, this possibility cannot be evaluated. Further research with more children cohorts across different languages is required to resolve this discrepancy.

### 2 Cognitive skills associated with lexical learning

Correlational analyses indicated a similar association between pseudo-word reading and lexical learning across languages. This is consistent with the self-teaching hypothesis according to which decoding is an important mechanism for lexical learning [27]. However, the fact that the Italian children are worse at lexical learning even when their decoding skills are as good or better than those of the English children supports previous studies showing that orthographic learning skills are partially independent of decoding skills [104].

As for cognitive predictors, our results show that phonological awareness and RAN indexes were both associated with lexical learning in English and Italian children. This was expected and confirms previous studies (*e.g*., [13, 19]). Both tasks measure the quality of phonological and orthographic representations [105–107] which is important to encode and retain a new orthographic representation. Additionally, RAN measures efficient sequential visual scanning and speed of lexical retrieval which may also be important for lexical learning. RAN, however, did not explain any further variance after the phonological awareness in the regression analysis. This might depend to the high shared variance between them but also to the fact that lexical learning was carried out without time limits reducing the need of fast lexical retrieval tapped by RAN.

The association between RAN/phonological awareness and lexical learning, however, does not mean that these tasks tap identical abilities. The ability to establish a new representation in a crowded lexical space by encoding a new ordering of phonemes or letters (i.e., lexical learning) requires skills that go beyond the ability to fast retrieve an already established lexical representation (RAN) and/or to manipulate it in phonological awareness tasks. As reviewed in the Introduction, results from the dyslexia literature strongly point to this independence. Within an adult dyslexic group, variation in reading and spelling abilities are *solely* explained by lexical learning, while phonological awareness is only associated with the ability to process novel words (see in particular [1, 3, 17]).

Together with similarities, we also found differences in the skills associated with lexical learning in the two languages. In the Italian children, even *written* lexical learning was associated with phonological STM, differently than in the English children, where there was no such association (coherently with [1]). By contrast, in English, lexical learning was more related to a task—the letter span task—which taps visual-attentional capacity. Moreover, tasks tapping visuo-serial attention and order letter encoding were more associated with lexical learning in Italian, than in English. Overall, these results indicate different strategies used by the Italian and English children to learn new written words which, in turn, may be related to their different experiences in learning to read and write, as we have described. The consistency of the Italian orthography may encourage phonological recoding which is supported by phonological STM and by serial visual attention which is used to bind individual letter identities to positions. By contrast, the inconsistency of the English orthography may encourage whole-word orthographic processing or, in any case, the processing of larger orthographic units for which good visuo-attentional capacity (the ability to process a chunk of orthographic input at once) is important.

Previous studies have underscored the importance of visuo-attentional skills for reading. Deficits in processing visual sequences are common among dyslexic children and adults with poor lexical processing [3, 59–60, 108–110]. In addition, our results suggest that different types of visual attention may be more important depending on the type of orthography to be learned. Visual-attentional capacity (tapped by a letter span task), may be more important for learning the orthography of inconsistent languages where focusing on larger processing units is more important [111;39]. Serial visual attention/letter order encoding (tapped by matching and order reconstruction tasks) is more important in the learning of consistent orthographies where a focus on smaller processing units is acceptable and/or desirable, especially in the early phases of acquisition.

Note that cross-linguistic differences in lexical learning might be mediated also by differences in the teaching methods used. Although in recent years a phonic approach has been increasingly used in English schools [112], teaching strategies still place a stronger emphasis on acquiring sight vocabulary in English than Italian. Therefore, the Italian children may be more accustomed to deploy sequential attention to processes individual letters in reading and spelling because grapheme-phoneme mapping are consistent even at this level. Instead, the English children may be more accustomed to process larger chunks of orthographic input because grapheme-phoneme mapping are inconsistent at the level of individual letters. These differences may give an advantage to the English children when having to learn whole new written as in our experimental task.

### 3 Study limitations

Some limitations of the present study should be pointed out.

First, as stated above, the characteristics of school entrance in the two countries are such that age could not be perfectly matched. Therefore a residual differences in age may have contributed to generate the observed findings. Additionally, cross-linguistic differences were detected also in some cognitive skills (e.g., in STM), that, in turn, might differently affect the performance at lexical learning task. However, when we carried ANCOVAs using the STM index as a covariate, the main findings of the study (and in particular all the language by learning trials interactions) were maintained in both age-matched and grade-matched comparisons.

Second, due to funding shortage we could carry out a grade-match comparison only for the older cohorts of children.

Finally, a number of limitations regard the experimental paradigm used.

- Our paradigm used a corrective feedback: this implies that, despite children were exposed the same number of times to the stimulus to be learnt, practice in spelling pseudo-words was greater for children that misspelled or omitted the pseudo-words because they were asked to spell them again. This means that the Italian participants–who were less accurate—received more practice. In spite of that, however, their showed a lower acquisition rate.
- In order to encourage encoding the stimulus as an orthographic representation and minimize phonological memorization, child did not have to read aloud the pseudo-word to be learn. This did not allow checking if the stimulus was read correctly. We think, however, that it is very unlikely that reading contributed to learning difficulties because in the introductory trial to the test all children were very accurate at reproducing the stimuli immediately after presentation.
- Given our task, we cannot be sure whether difficulties in learning involved more learning the linguistic stimuli or learning associations with pictures, but the type of errors made strongly suggest that association difficulties played a marginal role.
- Finally, one can note that our stimuli allowed phonologically plausible errors to be made in English but not in Italian (because English pseudo-words, but not Italian pseudo-words could be spelled in different, phonologically plausible ways). The Italian children, however performed more poorly in spite of this advantage.

## Conclusions

The ability to learn new words is at the core of human language. It is also fundamentally related to dyslexic difficulties because variation in this ability strongly predicts variation in dyslexic’s severity and outcomes in adulthood. While several studies have examined this skill in English, only a few have examined it in other more consistent orthographies (and, to this date, none that we know in Italian). Our study highlighted greater lexical learning skills in English children, learning an inconsistent orthography, compared to Italian children, learning a consistent orthography. Differences could depend on better storing and/or recalling larger orthographic representations in English than in Italian as showed by a stronger association with visuo-attentional capacity in English and with phonological STM and visuo-serial attention in Italian. Our results highlight how differences in orthographic consistency modulate learning strategies across languages.

## Supporting information

S1 Appendix Italian and English stimuli used for the lexical learning task. (DOCX)

S2 Appendix Italian and English pseudo-words used for the reading task. (DOCX)

S3 Appendix Pearson correlations between each predictor enter in the regression model, separately for English (low part of the table) and Italian children (high part of the table). Phon Awar = Phonological awareness; * p < .05; ** p < .*01*.
(DOCX)
