# Supplementary material for: The ability to learn new written words is modulated by language orthographic consistency
Source: PLoS One. 2020 Feb 13;15(2):e0228129. doi: 10.1371/journal.pone.0228129 (PMC7018089; doi:10.1371/journal.pone.0228129)
Supplement: S1 Appendix — (DOCX) [file pone.0228129.s001.docx]

## S1 Table. Italian and English stimuli used for the lexical learning task.

|  | | Picture name | phoneme length | word frequency | log word frequency |  | pseudoword | original word | log bigram frequency | letter length | N-size | N-size |
| --- | --- | --- | --- | --- | --- | --- | --- | --- | --- | --- | --- | --- |
| **VERSION FOR OLDER CHILDREN** | **ITALIAN** | unicorno | 8 | 0 | 0 |  | bolinco | valanga | 7.7 | 7 | 0 | 0 |
|  |  | camino | 6 | 7.3 | 0.9 |  | espilfo | asfalto | 7.3 | 7 | 0 | 0 |
|  |  | casco | 5 | 4.9 | 0.7 |  | stegli | spugna | 7.9 | 6 | 1 | 1 |
|  |  | busta | 5 | 17.6 | 1.2 |  | flicatta | flanella | 8.9 | 8 | 0 | 0 |
|  |  | pinguino | 8 | 0 | 0 |  | ogguira | agguato | 7.3 | 7 | 0 | 0 |
|  |  | pavone | 6 | 3.8 | 0.6 |  | togghia | secchio | 7.4 | 6 | 0 | 0 |
|  |  | vulcano | 7 | 7.8 | 0.9 |  | vontino | vendetta | 8.0 | 7 | 1 | 1 |
|  |  | dinosauro | 9 | 1.6 | 0.2 |  | calucia | valigia | 7.7 | 7 | 0 | 0 |
|  |  | robot | 5 | 5.7 | 0.8 |  | spaffo | staffa | 7.3 | 6 | 0 | 0 |
|  |  |  | 6.6 | 6.9 | 0.6 |  |  |  | 7.7 | 6.8 | 0.2 | 0.2 |
|  | **ENGLISH** | unicorn | 8 | 16.1 | 1.2 |  | ipalanche | avalanche | 8.0 | 9 | 0 | 0 |
|  |  | robot | 5 | 3.9 | 0.6 |  | stonte | sponge | 8.7 | 6 | 0 | 0 |
|  |  | volcano | 7 | 3.7 | 0.6 |  | olphand | elephant | 8.4 | 7 | 0 | 0 |
|  |  | dinosaur | 8 | 1.7 | 0.2 |  | grollen | flannel | 8.1 | 7 | 0 | 0 |
|  |  | chimney | 6 | 7.3 | 0.9 |  | entush | ambush | 8.1 | 6 | 0 | 0 |
|  |  | envelope | 7 | 19.5 | 1.3 |  | zackep | jacket | 7.7 | 6 | 0 | 0 |
|  |  | telephone | 7 | 3.6 | 0.6 |  | fingent | vengeance | 7.2 | 7 | 0 | 0 |
|  |  | helmet | 6 | 9.5 | 1 |  | lottube | lettuce | 8.6 | 7 | 0 | 0 |
|  |  | peacock | 5 | 3 | 0.5 |  | stannat | stirrup | 7.3 | 7 | 0 | 0 |
|  |  |  | 6.6 | 7.6 | 0.8 |  |  |  | 8.0 | 6.9 | 0 | 0 |
| **VERSION FOR YOUNGER CHILDREN** | **ITALIAN** | camino | 6 | 7.3 | 0.9 |  | flicatta | flanella | 8.9 | 8 | 0 | 0 |
|  |  | busta | 5 | 17.6 | 1.2 |  | ogguira | agguato | 7.3 | 7 | 0 | 0 |
|  |  | robot | 5 | 5.7 | 0.8 |  | togghia | secchio | 7.4 | 7 | 0 | 0 |
|  |  | unicorno | 8 | 0 | 0 |  | vontino | vendetta | 8.0 | 7 | 1 | 1 |
|  |  | pavone | 6 | 3.8 | 0.6 |  | calucia | valigia | 7.7 | 7 | 0 | 0 |
|  |  | vulcano | 7 | 7.8 | 0.9 |  | spaffo | staffa | 7.3 | 6 | 0 | 0 |
|  |  |  | 6.2 | 8.4 | 0.7 |  |  |  | 7.8 | 7.0 | 0.3 | 0.3 |
|  | **ENGLISH** | dinosaur | 8 | 1.7 | 0.2 |  | glollen | flannel | 8.1 | 7 | 0 | 0 |
|  |  | chimney | 6 | 7.3 | 0.9 |  | entush | ambush | 8.1 | 6 | 0 | 0 |
|  |  | envelope | 7 | 19.5 | 1.3 |  | zackep | jacket | 7.7 | 6 | 0 | 0 |
|  |  | telephone | 7 | 3.6 | 0.6 |  | fingent | vengeance | 7.2 | 7 | 0 | 0 |
|  |  | helmet | 6 | 9.5 | 1 |  | lottube | lettuce | 8.6 | 7 | 0 | 0 |
|  |  | peacock | 5 | 3 | 0.5 |  | stannat | stirrup | 7.3 | 7 | 0 | 0 |
|  |  |  | 6.3 | 8.9 | 0.8 |  |  |  | 7.9 | 6.7 | 0 | 0 |
