# Supplementary material for: The ability to learn new written words is modulated by language orthographic consistency
Source: PLoS One. 2020 Feb 13;15(2):e0228129. doi: 10.1371/journal.pone.0228129 (PMC7018089; doi:10.1371/journal.pone.0228129)
Supplement: S2 Appendix — (DOCX) [file pone.0228129.s002.docx]

**S2 Appendix.** Italian and English lists of pseudo-words (for each length subset N=10) used in the pseudowords reading task. For each stimuli set, the average number of phonemes and syllable is reported.

ITALIAN:

**4- letters:** Zila, Muci, Esfi, Siba, Libo, Bipo, Fipo, Mafe, Gomi, Urse (phonemes = 4.0, sd = 0; syllables = 2.0, sd = 0);

**5- letters:** Nisca, Bilfa, Sipio, Arsia, Bumbo, Libbe, Sinno, Ostra, Fisti, Fuago (phonemes = 4.9, sd = 0.6; syllables = 2.0, sd = 0);

**6- letters:** Matoba, Sibbio, Nulote, Minefa, Affime, Fietta, Deparo, Sancio, Espele, Chippo (phonemes = 5.4, sd = 0.7; syllables = 2.6, sd = 0.5);

**7-9- letters:** Reffina, Nagopio, Isterno, Soffatto, Ghioggia, Fibestre, Sipinzio, Ostedane, Firiglia, Bucchiole (phonemes = 6.8, sd = 1.0; syllables = 3.0, sd = 0.5).

ENGLISH:

**4- letters:** Sarn, Klib, Nard, Zale, Maly, Gilm, Leck, Zoof, Dant, Cois (phonemes = 3.5, sd = 0.5; syllables = 1.2, sd = 0.4);

**5- letters:** Glock, Ploth, Glame, Naver,Narvy, Flape, Cound, Panio, Stoln, Saple (phonemes = 4.2, sd = 0.4; syllables = 1.4, sd = 0.7);

**6- letters:** Vorner, Tranch, Daminy, Jumple, Retter, Zunder, Ollice, Dilber, Colune, Sinter (phonemes = 5.0, sd = 0.7; syllables = 2.0, sd = 0.5);

**7-9- letters:** Andicle, Clandet, Exandle, Huscant, Horping, Omidion, Dartyer, Bisbance, Gosbital, Tinection (phonemes = 6.6, sd = 1.0 ; syllables = 2.6, sd = 0.5).
