# Supplementary material for: The ability to learn new written words is modulated by language orthographic consistency
Source: PLoS One. 2020 Feb 13;15(2):e0228129. doi: 10.1371/journal.pone.0228129 (PMC7018089; doi:10.1371/journal.pone.0228129)
Supplement: S3 Appendix — Phon Awar = Phonological awareness; * p < .05; ** p < .01. (DOCX) [file pone.0228129.s003.docx]

S3 Table. Pearson correlations between each predictor enter in the regression model, separately for English (low part of the table) and Italian children (high part of the table).

| r di Pearson | | Italian children | | | | | | | |
| --- | --- | --- | --- | --- | --- | --- | --- | --- | --- |
|  |  | Pseudoword Decoding fluency | Ran I. | Phon Awar I. | STM I. | VAS | Serial Attent/Order Encoding I. | Visual memory |  |
|  | Pseudoword Decoding fluency |  | - .48** | - .45** | - .41** | - .23** | .00 | - .17* |  |
| English children | Ran I. | - .68** | .43** |  | .62** | .19** | .08 | .35** |  |
|  | Phon Awar I. | - .27* | .14 | .34** |  | .13 | .00 | .23** |  |
|  | Phon STM I. | - .64* | .45** | 055** | .29** |  | .06 | .24** |  |
|  | VAS | .10 | .01 | - .15 | .17 | .06 |  | .20** |  |
|  | Serial Attent/Order Encoding I. | .07 | - .04 | .06 | .20 | - .5 | .00 |  |  |
|  | Visual memory |  | - .48** | - .45** | - .41** | - .23** | .00 | - .17* |  |

I. = index; Phon = Phonological; Awar = awareness; Attent = Attention; VAS = Visual Attentional Span; ** p < .05; ** p < .01.*
